# Hematopoietic progenitor cells and interleukin-stimulated endothelium: expansion and differentiation of myeloid precursors

**DOI:** 10.1186/1471-2172-9-56

**Published:** 2008-10-01

**Authors:** Anja Moldenhauer, Gesche Genter, Andreas Lun, Gürkan Bal, Holger Kiesewetter, Abdulgabar Salama

**Affiliations:** 1 Institute for Transfusion Medicine, Charité – Universitätsmedizin Berlin, Germany; 2 Institute for Laboratory Medicine and Pathobiochemistry, Charité – Universitätsmedizin Berlin, Germany

## Abstract

**Background:**

Cytokine-stimulated endothelial cells (EC) propagate hematopoietic progenitor cell (HPC) expansion. However, the effects on the functional capacities of cultured progenitors have not been evaluated. HPC were assessed by flow cytometry, colony and cobblestone assays and long-term cultures (LTC) after culturing in the supernatant of EC stimulated by IL-1β, IL-3 or IL-6.

**Results:**

EC incubation with IL-6 did not improve cell expansion in comparison to non-stimulated EC supernatant, while the HPCs' phenotype and functional capacities were retained. In contrast, IL-1β and IL-3 stimulation resulted in a 10- and 100-fold increase in cell numbers with more than 90% of these cells being CD33(+). Plating efficiencies and LTC initiating cells were greatest in IL-6 supernatants, whereas the highest numbers of burst-forming units were observed using IL-3. IL-1β supernatants diminished the number of 5-week cobblestone-areas, whereas the number of 2-week cobblestone areas remained equal to freshly isolated HPC. Fewer 2-week cobblestones and greater amounts of 5-week cobblestones were observed with IL-6 and IL-3. Expanded progenitors from all interleukin conditions were further matured into functional granulocytes.

**Conclusion:**

IL-1β and IL-3 stimulated endothelium induces proliferation and differentiation of myeloid precursors, while IL-6 treatment induced a benefit of HPC survival.

## Background

During local inflammation, a cytokinetic firework initiated by cellular defense mechanisms includes the secretion of TNFα, interleukin-1, -3 and -6. These cytokines promote the release of endothelial factors which also attract hematopoietic progenitor cells (HPC) [1]. Therefore, the use of cytokine-stimulated endothelium as a hematopoietic feeder layer could be of great interest.

Several cellular immune reactions are triggered by interleukins (IL) with multiple impacts on lymphocytes, granulocytes and endothelial cells [2]. IL-1, for example, induces prostaglandin E2 and collagenase synthesis thereby activating the metabolism of polymorphnuclear neutrophils [3]. The secretion of endothelial granulocyte-macrophage colony-stimulating factor (GM-CSF) and granulocyte colony-stimulating factor (G-CSF) is further stimulated by IL-1β [4]. IL-3 in synergism with GM-CSF, on the other hand, controls the HPC differentiation into myeloid cells [5]. In synergism with IL-6, IL-3 also supports the proliferation of progenitors from human blasts [6]. Within the bone marrow niche, IL-6, which is also produced by vasulcar endothelial cells, propagates the differentiation of neutrophils [7]. Both, IL-6 and a recombinant form of its soluble receptor, the so-called hyper IL-6, enhance the SCF-induced expansion of hematopoietic progenitors [8] through gp130 signaling [9]. IL-6, a mediator of the acute phase response, is one of the most complex cytokines released at sites of injuries or infections [10], and many of its activities are shared by IL-1 [11]. On endothelial cells, IL-6 preferentially supports endothelial adherence of lymphocytes [10] and induces endothelial cells to proliferate [12] hereby enhancing angiogenesis [13].

Taken together, these three inflammatory stimuli induce the secretion of endothelial factors propagating the proliferation and differentiation of HPC. We previously demonstrated that endothelial cells (EC) stimulated by tumor necrosis factor alpha (TNFα) induce the generation of dendritic cells from CD34(+) HPC [14]. Here, we present data contributing to the influence of the supernatants from interleukin-stimulated endothelium on the proliferation and differentiation of HPC into granulocytes which highlights potential use of endothelial cells for the maintenance and maturation of blood cells.

## Results

### Cell expansion

**Figure 1:**
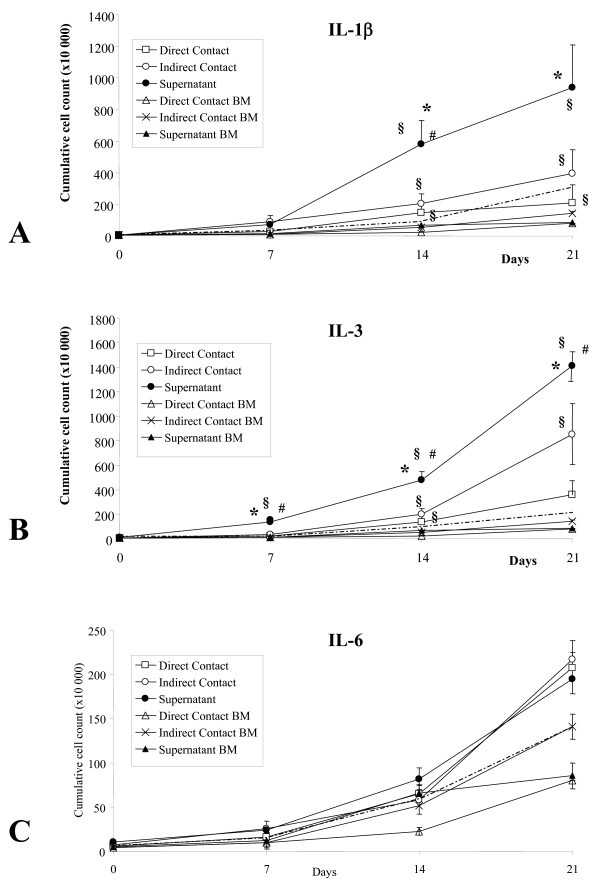
**Cumulative cell counts of proliferating progenitors in direct contact, non-contact and supernatant cultures**. Cell counts were determined by demi-depopulation after 7, 14 and 21 days and summarized. Culture conditions were as follows: A) HPC in direct contact with IL1-β stimulated EC (Direct Contact, *open squares*), on a 0.4 μm microporous transmembrane above the IL-1β stimulated EC (Indirect Contact, *open circles*), in the supernatant of IL-1β stimulated EC (Supernatant, *closed circles*), B) in direct contact with IL-3 stimulated EC (Direct Contact), on a 0.4 μm microporous transmembrane above IL-3 stimulated EC (Indirect Contact) and in the supernatant of IL-3 stimulated EC (*closed circles*). Significant differences to contact cultures (*), to indirect contact cultures (#) and to bone marrow (§ were only found in IL-1β and IL-3 dependent conditions. C) No significant differences were determined among the IL-6 stimulated EC culture conditions or among bone marrow fibroblast cultures. The HPC cell count at the beginning was 5.5 × 10^4 ^per 3 ml. Each point represents the average of at least three independent measurements. Bone marrow (BM) fibroblast cocultures consisted of direct contact (*open triangles*), indirect contact (*crosses*) and supernatant cultures (*closed triangles*). Dotted lines: HPC cultured in endothelial supernatants, to which IL-1β, IL-3 or IL-6 was added.

Direct contact between IL-β or IL3 stimulated EC and HPC significantly reduced the cumulative cell output as compared to non-contact and supernatant cultures (Figure 1). Stimulated supernatants led to two to three times higher cumulative cell counts than non-contact cultures (IL-3: 14.1 × 10^6 ^versus 8.5 × 10^6^; IL-1β: 9.3 × 10^6 ^versus 3.7 × 10^6^), which were twice as high as in direct contact cultures (IL-3: 3.6 × 10^6 ^and IL-1β:1.9 × 10^6^). Differences between IL-1β and IL-3 in cumulative cell numbers were not significant (p = 0.12). In IL-6 conditions, direct contact and supernatant conditions led to comparable cumulative cell counts (p > 0.13). Cell numbers in non-stimulated EC supernatant, to which single interleukins were added, had significantly lower cell counts in IL-1β and IL-3 conditions and lower cell numbers in IL-6 conditions, which was also the case, when HPC were cultured in endothelial plus stem cell medium including interleukins. IL-3 stimulated bone marrow fibroblasts led to significantly lower cumulative cell counts inducing on average a 15-fold cell expansion after two weeks. No significant differences were seen among different interleukins.

Since the highest cumulative cell numbers were achieved by culturing the HPC in stimulated endothelial supernatants, all further studies were preformed using these. Following a 7-days culture period, a minimum of 10-fold cell proliferation was observed in the supernatant of IL-1β and IL-3 stimulated endothelial supernatants (Table 1). After 14 days in culture, cell counts increased more than 140× with IL-1β, 83× with IL-3, and 6× in non-stimulated and in bovine serum albumin (BSA)-stimulated endothelial supernatants. Administration of IL-6 resulted in a five-fold increase in the cell number following two weeks in culture, which was equal to the fold increase of BSA- and non-stimulated endothelial supernatants (p > 0.13).

**Table 1:** Cell expansion in IL-stimulated endothelial supernatant following a period of 7 and 14 days and flow cytometric profile on day 7.

	**Concentration (U/ml)**	**Fold increases**		**Flow cytometry**
		**7 days**	**14 days**	CD33, 34, 45, 14, 16, 133
After isolation		N/A	N/A	

Control	Non-stimulated	1.2 ± 0.13	6.4 ± 1.2	
	0.1% BSA	1.3 ± 0.17	6.8 ± 1.6	

IL-1β	1	5 ± 1^a^	6.3 ± 0.12	N/A
	10	13 ± 2.2^a,b^	59.1 ± 13.3^a,b^	
	100	15.8 ± 2.5^a,b^	136.8 ± 23.3^a,b^	
	1,000	19.4 ± 8.6^a,b^	142.7 ± 23.5^a,b^	
	10,000	10.5 ± 2.8^a,b^	97 ± 22.4^a,b^	

IL-3	10	11.8 ± 1.6^a,b^	71.2 ± 10.5^a,b^	
	100	15.5 ± 1.6^a,b^	82.9 ± 14.5^a,b^	
	1,000	15.2 ± 1.4^a,b^	79.6 ± 9.9^a,b^	

IL-6	10	1.5 ± 0.51	2.8 ± 0.58	
	100	1.2 ± 0.22	5.2 ± 1.2	
	1,000	1.3 ± 0.21	4.6 ± 0.77	

Fold expansions were determined following a period of seven and fourteen days. Percentage of CD33, 34, 45, 14, 16 and CD133 positivity are depicted as circles (○: negative, less than 10%; quarter circle: 10 – 25% positivity; half circle: 25 – 50% positivity; ● more than 75% positive cells).

a: highly significant different compared to non-stimulated supernatant (p < 0.001); b: highly significant different compared to BSA supernatant (p < 0.001). Shown are mean results ± SE of twelve independent experiments. N/A: not applicable.

Optimum concentrations for IL-1β induced cell expansion were 100 and 1.000 U/ml, while IL-3 was observed to induce the highest cell numbers at 100 U/ml, though differences were not significant among different concentrations. Time-course observations demonstrated that IL-stimulation at varying concentrations (10, 100 and 1,000 U/ml) for 16 hours provided the highest increase in cell numbers as compared to 2, 4, 8, 24 and 48 hours.

### Characteristics of expanded cells

**Figure 2:**
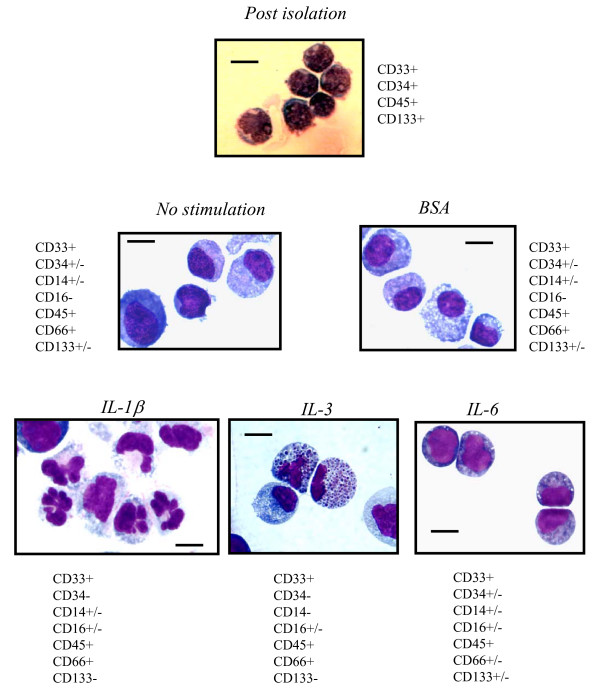
**Cytospin preparations of freshly isolated HPC and following culture for two weeks in non-stimulated, BSA or IL-stimulated EC supernatant**. Freshly isolated HPC *(Post isolation) *with a dense nucleus and small cytoplasmatic rim increased up to two-fold in size and gained cytoplasma in non-stimulated and BSA-stimulated supernatants. With IL-1β stimulated supernatant they developed into hypersegmented cells and also into monocytic cells in part, with an increase in cytoplasma content. More than 50% of the cells stimulated with IL-3 developed eosinophilic granula, whereas cells in IL-6 stimulated supernatant resembled freshly isolated cells. Cells cultured in IL-6, BSA- and non-stimulated supernatants were still positive for CD34 and CD133. Diffquik staining, size bar 1 μm. magnifications ×200. One representative result of twelve independent experiments.

More than 93% of the freshly isolated cells were positive for CD34, CD33 and CD45. The latter two remained highly positive following a period of two weeks in all of the culture conditions analyzed. When cultured with IL-1β or IL-3-stimulated supernatant, expanded cells lost the CD34 antigen following a one week culture period (Table 1). In contrast, on average 34.8 ± 6.7% of the cells cultured in BSA, IL-6 or non-stimulated supernatant stained positive for CD133, and 17.7 ± 5.2% were still CD34 positive in IL-6 induced supernatant. Although the loss of CD34 antigen was paralleled by a loss of CD133, a subset of CD34(-) cells retained the CD133 glycoprotein (see additional file 1)). Following a two week culture period, half of the cells in the IL-1β stimulated EC supernatant were CD16(+), and 15–25% of the cells carried the monocytic marker CD14 (Figure 2). Other glycoproteins tested were CD15 and CD19, which were rarely present in freshly isolated CD34 cells and did not increase upon culturing.

The receptor repertoire matched the observed changes in morphology. IL-1β and IL-3 generated supernatant induced a rather versatile morphology consisting of macrophage and granulocytic precursors with eosinophilic granula in case of IL-3 (Figure 2). In contrast, cells cultured in IL-6 stimulated EC mostly resembled freshly isolated HPC with round nuclei and low cytoplasmatic content. Cells expanded in non-stimulated or BSA supernatant increased slightly gaining little cytoplasm.

### Hematopoietic potential of expanded cells

#### Colony formation

A concentration-dependent increase of BFU-E colonies were determined in the cells cultured in supernatants from IL-1β stimulated EC. BFU-E were significantly higher than in the non-stimulated supernatants (p < 0.05, Table 2), in freshly isolated HPC or in those expanded in BSA stimulated EC supernatants (p < 0.035 at IL1β concentration of 1,000 U/ml). Here, the numbers of CFU-GM and mixed colonies were comparable to those observed post-isolation, but the plating efficiencies (PE) were the lowest being significantly lower than in freshly isolated HPC (p < 0.001).

**Table 2:** Colony forming activity of HPC expanded in IL-stimulated EC supernatant for one week.

	**Concentration (U/ml)**	**BFU-E **(×10^3^)	**CFU-GM **(×10^3^)	**CFU-Mix **(×10^3^)	**PE **(%)
Post isolation (5 × 10^4 ^cells)	N/A	2.5 ± 0.18	3.3 ± 0.48	0.16 ± 0.02	7.9 ± 0.57

Controls					
- No stimulus	N/A	2 ± 0.28	2.7 ± 0.46	0.36 ± 0.14	6.4 ± 0.67
- BSA	0.1%	3 ± 0.46	4.3 ± 0.74	0.14 ± 0.05	4.5 ± 1.1^c^

IL-1β	10	3.6 ± 1.1^a^	1.8 ± 0.56	0.16 ± 0.07	0.97 ± 0.06^a,b,c^
	100	5.2 ± 1.6^a,c^	2.4 ± 0.82	0.22 ± 0.08	1 ± 0.06^a.b.c^
	1,000	6.4 ± 1.2^a,b,c^	2.4 ± 0.43	0.22 ± 0.08	1.1 ± 0.08^a,b,c^

IL-3	10	10 ± 3.3^a,b,c^	3.1 ± 1	2.2 ± 0.74^a,b,c^	3.9 ± 1.3^c^
	100	9.5 ± 3.2^a,b,c^	3.1 ± 1	2.1 ± 0.71^a,b,c^	1.6 ± 0.54^a,c^
	1,000	12.8 ± 4.3^a,b,c^	6.3 ± 2.1^a,c^	5 ± 1.7^a,b,c^	1.7 ± 0.58^a,c^

IL-6	10	1.5 ± 0.34^c^	1.5 ± 0.37^b>,c^	0.28 ± 0.11	8.2 ± 1.7
	100	2.1 ± 0.4	3.1 ± 0.67	0.18 ± 0.07	8.9 ± 1.4
	1,000	3.1 ± 0.58	4 ± 0.69	0.24 ± 0.08	11.8 ± 1.2^a,b,c^

Hematopoietic colony formation was determined after fourteen days in semisolid methylcellulose cultures supplemented with erythropoietin, GM-CSF, IL-3 and stem cell factor. Total colonies were defined by multiplying counted colonies with the number of expanded cells divided by the number of input cells. Mean values ± SE from four to nine independent experiments conducted in triplicate. BFU-E: burst-forming unit erythrocyte; CFU-GM: colony-forming unit granulocyte macrophage; CFU-Mix; mixed colony-forming unit (granulocyte, erythrocyte, megakaryocyte, macrophage); PE: plating efficiency; N/A: not applicable; BSA: bovine serum albumin

a: significant different compared to non-stimulated supernatant; b: significant different compared to BSA supernatant; c: significant different compared to freshly isolated CD34(+) cells.

Significantly decreased plating efficiencies were also found in HPC expanded in IL-3 conditioned medium (p < 0.05). The values obtained were comparable to those in BSA-stimulated medium, but lower than those in naïve EC supernatant at concentrations of 100 and 1.000 U/ml IL-3 (p < 0.02). With IL-3, the highest overall numbers of BFU-E and mixed colonies were determined with BFU-E numbers three to five times, and CFU-Mix numbers 15 – 40 times higher than in cells post-isolation (p ≤ 0.025).

The highest plating efficiencies of all conditions tested were observed in cells cultured with IL-6 stimulated EC supernatant. At a concentration of 1,000 U/ml, plating efficiencies were two-fold higher than in cells cultured with non- or BSA-stimulated EC supernatant (p < 0.0026) and even significantly higher than in freshly isolated cells (p = 0.002). Compared to the latter group, the total numbers of BFU-E and CFU-GM were significantly lower at IL-6 concentrations of 10 U/ml (p = 0.005), but normalized at IL-6 concentrations of 100 U/ml and higher (p > 0.2).

#### CAFC and LTC-IC

The highest numbers of 2-week cobblestone area-forming cells were achieved following culture of HPC in IL-1β stimulated supernatant. At a supraphysiological concentration of 10,000 U/ml, approximately four times more 2-week cobblestones were found than in cells post isolation and twice as many as in those cultured in BSA-stimulated supernatant (p < 0.05) indicating the expansion of predominately myeloid progenitors (Table 3). The number of 2-week CAFC were comparable to freshly isolated HPC (p > 0.36) and those grown in BSA-stimulated EC (p > 0.1) at all other IL-1β concentrations. The highest numbers of 5-week CAFC, a parameter of the undifferentiated progenitors, were observed in cells which had been cultured in supernatants from IL6-, BSA- or non-stimulated EC. These CAFC figures were the only ones observed to be equivalent to those of freshly isolated HPC (IL-6: p > 0.095; BSA: p = 0.42; non-stimulated: p = 0.21). The highest numbers of LTC-IC were found in cells cultured in non-stimulated endothelial supernatant followed by freshly isolated CD34(+) cells and cells cultured in BSA- or IL-6 stimulated supernatants. Differences among these four groups were insignificant (p > 0.15). Significantly lower values were determined in cells expanded in 1,000 U/ml IL-1β-stimulated EC (p < 0.037), and those expanded in IL-3-stimulated EC (p < 0.025).

**Table 3:** Cobblestone area and long-term culture initiating cells (LTC-IC) of HPC post-isolation and of cells cultured in IL-stimulated EC supernatant for one week.

	**Concentration (U/ml)**	**CAFC**		**LTC-IC**
		**2-week**	**5-week**	
Post isolation	N/A	4.9 ± 0.73	23.2 ± 4.2	16.3 ± 3

Control	No addition	11.6 ± 2.5^c^	16.8 ± 4.5	17 ± 4.3
	0.1% BSA	9.6 ± 2.8	17.8 ± 4.5	16.2 ± 4.2

IL-1β	10	3.7 ± 1.1	5.4 ± 2.4^a,c^	7 ± 2.2
	100	4.9 ± 0.95	3.4 ± 1.2^a,b,c^	6.6 ± 2.3
	1,000	5.6 ± 0.92	0.45 ± 0.2^a,c^	3.7 ± 2.2^c^
	10,000	19.2 ± 7.1^b,c^	0.38 ± 0.17^a,b,c^	n.d.

IL-3	10	3.6 ± 1.1^a,c^	8.1 ± 2^a,c^	3.2 ± 2^c^
	100	4.3 ± 1.5^a^	2.8 ± 0.58^a,b,c^	2.3 ± 0.59^c^
	1,000	2.2 ± 0.5^a,c^	4 ± 0.88^a,b,c^	1.8 ± 0.61^c^

IL-6	10	3.6 ± 1.1	9 ± 2	11 ± 3.2
	100	4.3 ± 1.5	20.9 ± 4.1	13.2 ± 6.3
	1,000	2.2 ± 0.5^a,b^	15.7 ± 3.3	8.5 ± 3.4

Freshly isolated and expanded HPC were cultured on the murine bone marrow stroma cell line MS-5 and scored for cobblestone-area formation after two and five weeks. LTC-IC were scored by replating 5-week CAFC in methylcellulose for secondary colony formation. Shown are mean results ± SD of three independent experiments in triplicate; a: significant different compared to non-stimulated supernatant (p < 0.05); b: significant different compared to BSA supernatant (p < 0.05); c: significant different compared to freshly isolated CD34(+) cells.

### Granulocytic features and function of differentiated cells

**Figure 3:**
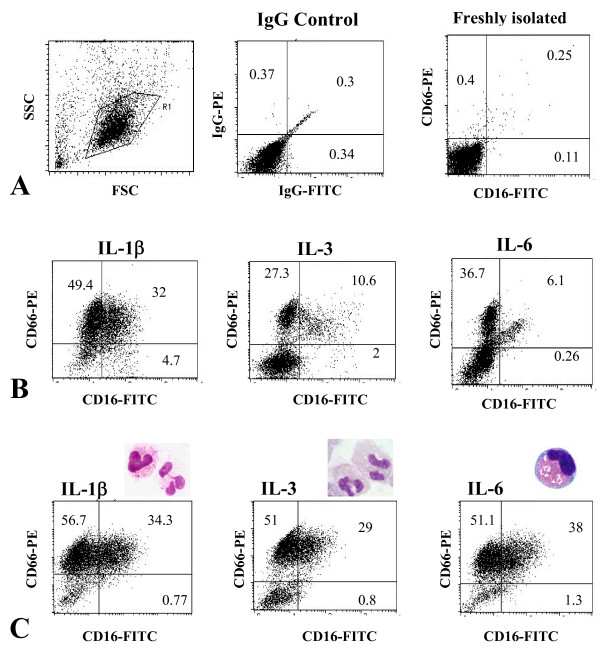
**Flow cytometry of expanded cells before and after culturing for a subsequent week in G-CSF**. Expression of CD16 and CD66 was up-regulated in HPC expanded in IL-3 and IL-6 stimulated EC cultures (p < 0.05), while in IL-1β cultures, no further up-regulation was observed. Increase of granulocytic glycoproteins occurred in parallel to the development of granulocytic morphology. Pictures were taken from one representative result of six independent experiments. A) forward scatter – side scatter, IgG control; B) CD16 and CD66 expression before culturing with G-CSF; C) CD16 and CD66 expression and cell morphology after culturing with G-CSF.

Extension of the cell culture for an additional week with G-CSF induced the up-regulation of the granulocytic markers CD16 and CD66 in all three interleukin conditions (Figure 3). Prior to G-CSF addition, only cells cultured in IL-1β-stimulated endothelial supernatant already had a high frequency of CD16 and CD66 positive cells, which was further increased following the addition of G-CSF. Thereafter, the cells also became highly positive for CD15, CD11b and CD11c. Control granulocytes differentiated in stem cell medium plus cytokines in the absence of endothelial supernatant developed an equivalent morphology and immunephenotype. There were no differences between the burst activities of G-CSF matured granulocytes from different interleukin conditions (p > 0.05, Table 4).

**Table 4:** Burst activities of differentiated cells expanded in IL-stimulated endothelial supernatant.

	**Control**	**E. coli**	**fMLP**	**PMA**
IL-1β (FBS)	52.5 ± 13.1 (593 ± 156.9)	509.5 ± 107.9 (169.1 ± 30.6)	62.7 ± 10.5 (1336 ± 320.5)	350.4 ± 95.5 (2873.4 ± 615.9)
IL-3	55.4 ± 13.2	800.7 ± 343.1	74 ± 33.1	784.3 ± 334.7
IL-6	39 ± 9.4	467.6 ± 167.31	177 ± 124	287.1 ± 104.5

After culturing the cells for one week with G-CSF (100 ng/ml) and keeping them overnight in human serum, expanded cells showed a ten-fold increased burst activity in response to E. coli and PMA. Oxygen radical formation in cells from different interleukin conditions were comparable (p > 0.05). Shown are average results of mean fluorescence activities ± SE from five independent experiments. In brackets: mean results after storage in FBS-based medium (n = 10).

PMA: phorbol 12-myristate 13-acetate; E. coli: Escherichia coli; fMLP: N-formyl-methionyl-leucyl-phenylalanin.

**Figure 4:**
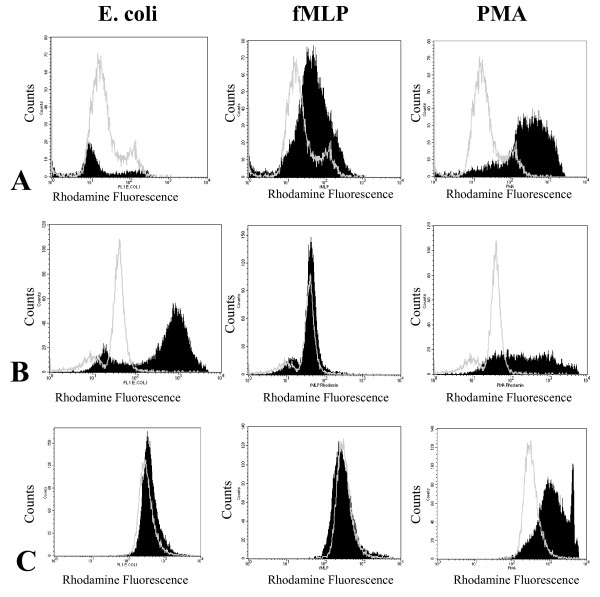
**Granulocytic functionality**. Phagoburst results are shown in response to PMA, fMLP and E. coli of HPC expanded in IL1-stimulated EC and following further differentiation by G-CSF in comparison to granulocytes differentiated by cytokines alone. A) HPC differentiated following expansion in IL1-stimulated EC supernatant; B) HPC differentiated following expansion in IL1-stimulated EC supernatant and overnight storage in human serum prior to analysis; C) HPC differentiated in a cytokine combination of erythropoietin, SCF and G-CSF without endothelial supernatant. Shown is one representative result of eight independent experiments. Shaded histograms: sample fluorescence; white line: negative control. PMA: phorbol 12-myristate 13-acetate; E. coli: Escherichia coli; fMLP: N-formyl-methionyl-leucyl-phenylalanin.

Differentiated cells were analyzed for their granulocytic function. Cells which were harvested directly from G-CSF cultures had high spontaneous burst rates, which were even higher than after they had been exposed to Escherichia (E.) coli (Figure 4A). Yet, these cells responded two- and ten-fold better to N-formyl-methionyl-leucyl-phenylalanin (fMLP) and phorbol 12-myristate 13-acetate (PMA), respectively. When the differentiated cells were incubated overnight in human serum at 37°C, E. coli or PMA induced a ten-fold burst, whereas no effect was seen in response to fMLP (Figure 4B). Burst rates between cells, which had been stored overnight in human serum and those without serum incubation were significantly different (p ≤ 0.018). Oxygen radical formation was also significantly higher in granulocytes generated in stimulated EC supernatant than in granulocytes differentiated with cytokines alone (Figure 4C), but lower than in granulocytes from peripheral blood.

## Discussion

Human endothelium, the gatekeeper between blood and tissue, plays a decisive role in the initiation of cellular immune responses [3]. The way in which endothelium influences HPC in the blood circulation during an inflammation, however, is unknown. The data presented here gives new insights into the unique role of endothelium as a conductor in the inflammatory orchestra, especially on the influence of IL-1β, IL-3 and IL-6 stimulated endothelium on the proliferation and differentiation of HPC.

The highest fold increases were determined in supernatants from IL-1β-stimulated EC. IL-1, for example, does induce endothelial cells to secrete hematopoietic growth factors [15] like stem cell factor [16], GM-CSF [17] and G-CSF [18]. The latter two are well-known to be responsible for HPC expansion and granulocytic differentiation. In fact, Bioplex assays confirmed the IL-1β induced increase of G-CSF, GM-CSF, IL-1, IL-6 and IL-8 which are known hematopoietic growth factors [19]. IL-13, IL-17, macrophage inflammatory protein 1 and monocyte chemoattractant protein 1 were also higher in IL-1β stimulated EC supernatant than in BSA-stimulated samples. This could explain why predominately white blood cell precursors expanded in IL1β-conditioned EC medium retaining CD33, a marker for myeloid progenitors. Functional tests proved the proliferation of myeloid progenitors resulting in high numbers of 2-wk cobblestones and the lack of primitive HPCs demonstrated by the absence of 5-wk CAFC and LTC-IC.

One effect of IL-1β on HPCs is the indirect enhancement of their sensitivity for IL3 [6], possibly by upregulating IL-3 receptors on endothelial cells. IL-3 improves the ex vivo expansion of HPC induced by FLT3/FLK2-ligand, stem cell factor and thrombopoietin [20]. In our culture system, IL-3 led to an equivalent fold increase of cell numbers as IL-1β and the highest number of mixed colonies, which speaks in favor of the expansion of oligopotential HPC. The reduced number of 5-week cobblestones and long-term culture initiating cells, however, opposes the expansion of primitive hematopoietic stem cells. Administered on endothelial cells, IL-3 induces the *in vitro *adhesion of basophilic granulocytes [21] with endothelium supporting the IL-3 dependent differentiation of eosinophilic granulocytes [22]. The latter stands in agreement with our morphologic results showing the development of eosinophilic granula in expanded HPC.

Another supporter of the IL-3 dependent HPC proliferation is IL-6 [23]. Previous works analyzed the importance of IL-6 within the hematopoietic/endothelial conundrum. For example, IL-6 was found to be one of the most crucial endothelial factors supporting HPC expansion in a combination of multiple cytokines plus endothelial cells [24]. More committed cells do express the receptor for IL-6 [25], whereas it is absent on early uncommitted HPC, although these cells are responsive to IL-6 in complex with the soluble IL-6 receptor [8,26]. Their combined use dramatically stimulates the expansion of primitive hematopoietic progenitor cells in the presence of SCF [8,26]. This might account for the observed delay in cell expansion, which led to a five-fold increase one week later than in IL-1β and IL-3 endothelial supernatants.

In our study, HPC maintained in IL-6 stimulated EC supernatant retained CD34 and CD133, which was also the case in BSA- and non-stimulated cultures. Besides, cells grown in supernatants from IL-6, BSA or non-stimulated EC had the best plating efficiencies, the highest number of 5-week cobblestones and LTC-IC indicating that mainly primitive progenitors expanded. Considering the fold increases in BSA- and non-stimulated supernatant, one could hypothesize that IL-6 had no effect on the endothelial cells despite STAT3 phosphorylation. However, from the five conditions tested, only cells grown in IL-6-stimulated EC supernatant had a significantly higher plating efficiency than freshly isolated HPC. Therefore, IL-6 seemed to induce the secretion of endothelial factors propagating the expansion of hematopoietic progenitors, whereas IL-1β and IL-3 induced the secretion of endothelial factors promoting the proliferation of myeloid precursors. In former studies [27], IL-6 could only affect endothelial chemokine production in the presence of soluble IL-6 receptor. As we used fetal and human bovine serum in our culture conditions, the soluble IL-6 receptor was probably drawn from the applied media supplements.

The add-back of interleukins to non-stimulated EC conditioned medium did not significantly influence cell expansions compared to non-stimulated supernatant which speaks against a contaminating interleukin effect. Intriguingly, non-stimulated and BSA-generated supernatants also induced the proliferation of HPC, although at much lower levels. BSA stimulation actually increased endothelial G-CSF, GM-CSF, IL-6 and IL-8, though the levels were much lower than in IL-1β stimulated supernatants (unpublished data). Following a period of two weeks, fold increases were equivalent to those determined in IL-6 conditioned medium, and the results of CAFC in combination with LTC-IC suggest that the expansion of undifferentiated HPC was initiated. This stands in line with other studies demonstrating that endothelial cells support HPC survival and expansion [14,28,29]. As co-infusion of bone marrow mesenchymal cells with bone marrow HPC supports engraftment of bone marrow transplants [30], simultaneous application of human umbilical cord EC with cord blood-derived HPC could improve the survival of cord blood grafts. Accordingly, cerebral endothelial cells were found to be very promising adjuvants for bone marrow regeneration in animal studies [31]. Human umbilical cords, a much more accessible source of endothelial cells, could be used in the same way, being isolated whenever cord blood is collected.

In the absence of interleukins, more progenitors expanded, if they were cultured in direct contact with EC. When interleukins are added, however, a different scenario opens. Like Jazwiec and colleagues we found a higher cell expansion, if HPC and EC were cultured separately from each other [4]. This implies that ligand-receptor interactions between both cell types prevents HPC proliferation. Another reason could be that endothelial cells reabsorb hematopoietic growth factors in a paracrine-autocrine fashion [32], thereby competing with the HPC for growth factor internalization and consumption. Since endothelial cells are positive for c-kit, the receptor for stem cell factor, as well as GM- and G-CSF receptors [33,34], this could very well be the case.

Expanded cells from all IL culture conditions could be differentiated into functionally mature granulocytes with typical granulocytic immunephenotypes and burst activities double as high as of HPC grown in cytokines alone. Although high reactions of cells generated in IL-stimulated medium were determined in response to PMA and fMLP, oxygen bursts in response to E. coli were initially below the negative control. This was related to high spontaneous burst rates, which disappeared, if the cells had been incubated in human serum overnight. That does imply that the culture medium contained fluorescent components which needed to be washed out before challenging the cells. One also has to bear in mind, that bacterial toxins and PMA are known as strong stimulators of ADAM17, a protease which is responsible for the shedding of various cell proteins like TNFα and the soluble IL-6 receptor [35,36]. Once transfused in vivo, these cells might therefore be functionally competent.

In bioengineering, the use of feeder layers has been repeatedly recommended for HPC expansion [37-41]. Coculture models usually include the administration of stroma cells [42,43], while other groups focus on the application of endothelial cells [28,44,45].

In concordance with previous results on TNFα-stimulated EC [14], IL-stimulated bone marrow fibroblasts did not lead to the same fold increases as endothelium. In contrast to findings from other groups [46-48], IL-3 stimulated bone marrow fibroblasts led to significantly lower cumulative cell counts. One reason could be the fact that we used only single cytokines and not a combination of hematopoietic growth factors. Another could lie in the different sources of stroma cells [49]. We used either primary bone marrow stroma cells isolated from leukemic patients or the murine stroma cell line MS-5. The latter is known to support the expansion of primitive hematopoietic progenitor cells [50] in the absence of growth factors [51]. Human interleukins don't necessarily have to have a stimulatory effect on these. Stroma cells from leukemic patients are subjected to several variables like patient's age, stage of disease or therapeutic regime, which can account for an abnormal milieu in cell cultures. Though EC from human umbilical cords also vary interindividually, they still are of comparable quality.

Since endothelial cells and cord blood HPC can be isolated from the same donor simultaneously, cytokine stimulated EC could be used in autologous bioreactors for the expansion and differentiation of homologous blood cells. This distinguishes endothelial cells as an attractive feeder population, permitting spatial separation of feeder and expanding cells. As supernatants of IL-stimulated EC led to higher fold increases as contact and indirect contact cultures, sequential instead of simultaneous culturing is possible starting with endothelial cell plating and harvesting of supernatants followed by HPC expansion cultures.

In conclusion, supernatants from interleukin-stimulated endothelial cells can be used to expand and differentiate hematopoietic cells *ex vivo*. While IL-6 helped to preserve HPC functionality, IL-1β and IL-3 rather induced the differentiation of granulocytic precursors. Further genetic analyses, e.g. by oligonucleotide microarrays of stimulated and non-stimulated EC could further clarify which factors involved in HPC expansion and/or differentiation are produced by endothelial cells.

## Methods

### Cord blood, HPC isolation

Cord blood specimens were collected in heparin-coated syringes and blue caps from full-term delivered neonates, following written consents from the mothers. Mononuclear cell fractions were isolated by Ficol (Biochrom, Berlin, Germany) followed by two wash steps. CD34(+) HPC were immunomagnetically selected as previously described [52].

### IL-stimulation of endothelial and bone marrow stroma cells

Human umbilical cord EC were obtained by flushing umbilical veins with 0.1% collagenase (Sigma-Aldrich, Steinheim, Germany) [14]. The cells were then cultured in endothelial cell conditioning medium consisting of M199 (Biochrom, Berlin, Germany) supplemented with 16% fetal bovine serum (FBS, Hyclone, South Logan, UT), 4% human serum from healthy volunteers, 2 mM L-glutamine, 0.15 mg/ml endothelial growth factor supplement (Intracel; Rockville, MD), 0.015 mg/ml heparin and 1% fungicide. Bone marrow stroma cells were harvested from bone marrow aspirates from leukemia patients [14] and cultured in RPMI 1640 supplemented with 10% FBS, 2 mM L-glutamine and 100 U/ml penicillin/streptomycin. In two experiments, cells from the murine bone marrow line MS-5 (kindly provided by Katja Weisel, Germany) were used. Confluent monolayers from passages two to six were stimulated with either IL-1, -3 or -6 (all Peprotech, Rocky Hill, MD) for 16 hours. All cytokines were dissolved in 0.01% BSA and phosphate buffered saline (PBS). Supernatants were filtered through a 0.2 μm sterile filter and diluted 1:2 with stem cell medium. Stem cell medium consisted of Iscove's modified Dulbecco's medium (IMDM, Biochrom) supplemented with 20% FBS, 2 mM L-glutamine, 50 μg/ml gentamicin and 7.3 × 10^-5 ^M mercaptoethanol. Optimum duration of interleukin-stimulation was evaluated in time-course experiments (2, 4, 8, 16, 24 and 48 hours).

### HPC and EC Culture Systems

In supernatant samples, CD34(+) hematopoietic progenitor cells (10^4^-10^5 ^cells in 3 ml) were cultured in a 1:2 mixture of IL-1β, IL-3 or IL-6 stimulated EC supernatant and stem cell medium in 6-well culture plates. Interleukin concentrations for endothelial stimulations ranged from 1 to 10,000 U/ml. Control samples consisted of non-stimulated supernatant and supernatant stimulated with 20 μl of 0.1% bovine serum albumin (1 μg/mL, Sigma-Aldrich) leading to a final concentration of 10 ng/ml. Other controls consisted of non-stimulated endothelial supernatant mixed with stem cell medium and supplemented with IL-1β, IL-3 or IL-6 as well as a mix of endothelial and stem cell medium supplemented with interleukins. Cultures were fed once to twice a week by removal of 0.5 ml and replacement with 0.7 ml supernatant-media-mix.

Cell counts, morphology, immune phenotype and colony formation were determined following a period of one and two weeks. Initial experiments included the comparison of direct contact and indirect contact systems. Here, HPC (10^4^-10^5^) were either cultured in direct contact with a confluent EC monolayer or on top of a 0.4 μm microporous transmembranes (Corning costar, ) above the EC layer. On five occasions, endothelial cells were replaced by bone marrow fibroblasts.

### Cell counts, morphology and flow cytometry

After seven, fourteen and, for cumulative cell counts, after 21 days viable cells were determined by a hemocytometer using trypan blue. In direct contact cultures, HPC were distinguished from EC by assessing the number of CD45(+) cells by flow cytometry.

Frequencies of CD14, CD15, CD16, CD19, CD33, CD34, CD45, CD66 (all BD Pharmingen, San Diego, CA) and CD133 (PE-labeled, Miltenyi Biotech, Bergisch-Gladbach, Germany) positive cells were measured by dual staining as described previously [53]. Briefly, 0.5 – 1 × 10^5 ^cells were washed once with 1 ml PBS, and resuspended in 100 μl plus 1.8 μl anti-human FITC or PE labeled antibodies. After incubation for 20 minutes at 4°C, excess antibodies were removed and stained cells were analyzed by flow cytometry (FACScan, Becton Dickinson, Heidelberg, Germany).

Light microscopy of cytospin preparations were carried out by Diffquik staining [54], and pictures were taken by a SC 35 Type 12 camera (Olympus, Hamburg, Germany) at 40× magnification.

### Hematopoietic colony formation

The plating efficiency of the isolated HPC was analyzed by plating 1 × 10^3 ^CD34(+) hematopoietic progenitor cells in 1 ml of methylcellulose (Stem cell Technologies, Vancouver, BC) supplemented with 30% fetal calf serum, 20 ng/ml c-kit ligand (stem cell factor, Peprotech), 20 ng/ml IL-3, 6 U/ml erythropoietin (Roche, Hertfordshire, GB) and 100 ng/ml granulocyte-macrophage (GM) colony-stimulating factor (CSF, Peprotech) [52]. Input numbers of cultured cells were adjusted by multiplying 10^3 ^with the fold increases. After two weeks, cultures were scored for granulocyte-macrophage colony-forming units (CFU-GM), mixed colony forming units (CFU-Mix) and burst-forming units erythrocyte (BFU-E). Colonies consisting of more than 50 cells were scored using an inverted microscope and the plating efficiencies were determined by dividing the total number of colonies by the number of input cells. Each measurement was performed in triplicate.

### Cobblestone area-forming cells (CAFC) and long-term culture initiating cells (LTC-IC)

CAFC assays were performed as previously described [55]. In brief, appropriate numbers of freshly isolated or expanded cells were seeded onto confluent murine bone marrow MS-5 stroma in 12.5-cm^2 ^flasks in α-MEM medium supplemented with 12.5% horse serum (PAA Laboratories, Pasching, Germany), 12.5% FBS, 10^-5 ^M hydrocortisone, 2 mM L-glutamine, 50 μg/ml gentamicin, and were demi-depopulated on a weekly basis. Cobblestone areas were scored at two and five weeks using an inverted phase microscope to identify phase-dark hematopoietic areas of at least five cells beneath the stromal layer. The LTC-IC content was determined by assaying for secondary colony forming cells in subsequent methylcellulose cultures following five weeks of stromal co-culture.

### Granulocytic differentiation

For granulocytic maturation, two-week expanded cells were cultured for an additional week in IMDM supplemented with 20% FBS and 100 ng/ml G-CSF (4 × 10^5 ^cells in 2 ml). In some experiments, cells were kept at 37°C in autologous or pooled human serum prior to their functional assessment. Oxygen radical formation was determined using the commercially available Phagoburst test (Orpegen, Heidelberg, Germany) as recommended by the manufacturer [56]. Briefly, cultured cells were subjected to external stimuli such as opsonized E. coli, fMLP or PMA. Samples without any additional stimulus served as negative control. Dihydrorhodamine 123 (fluorescent rhodamine) indicated the presence of free oxygen radicals, which corresponded to NADPH oxidase activity. Cells were gated on granulocytes and their rhodamine fluorescence was measured by flow cytometry.

### Statistical analysis and ethics

Student's t-tests for paired samples to compare results from interleukin- and non-treated or BSA-treated EC, calculation of means, standard errors and p-values were performed using Microsoft Excel 2000, Version 9.0. Differences with p-values less than 0.05 were termed as significant. The study was approved by the ethical review board of the Charité, registration number EA1/012/08.

## Authors' contributions

AM designed the study, initiated the experiments, performed the statistical analysis and drafted the manuscript. GG carried out the cell cultures, flow cytometries, colony and cobblestone assays. GB performed the add-back experiments. AL performed granulocyte functional assays. HK conceived of the study and provided intellectual and financial support. AS conceived of the study, helped in the design and coordination and helped to draft the manuscript. All authors read and approved of the final version.

## Supplementary Material

Additional file 1 CD133 and CD34 expression of HPC cultured in IL-6 stimulated endothelial supernatant. after one week in culture (A) CD133 and CD34 were still present, whereas a distinct subset of CD133(+) cells did not stain for CD34. Reduced CD34 was paralleled by reduced CD133 positivity in the second week (B), while still more CD133(+)CD34(-) cells than double positive cells were detected. Click here for file
