# Supplementary figures and images for: Hematopoietic progenitor cells and interleukin-stimulated endothelium: expansion and differentiation of myeloid precursors
Source: BMC Immunol. 2008 Oct 1;9:56. doi: 10.1186/1471-2172-9-56 (PMC2570655; doi:10.1186/1471-2172-9-56)

**A**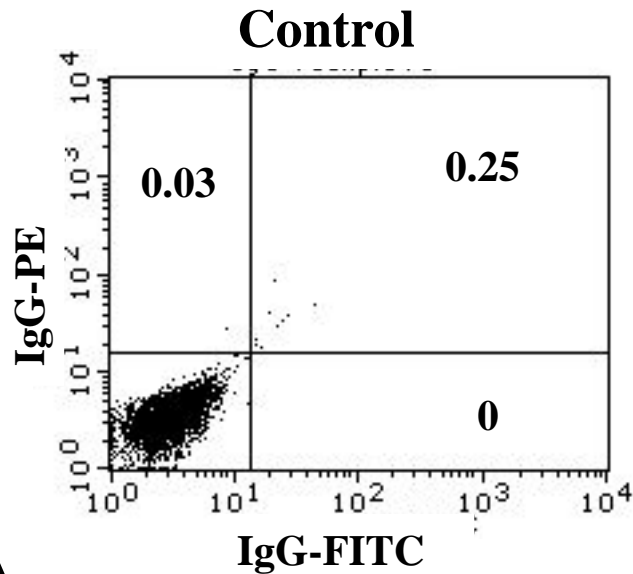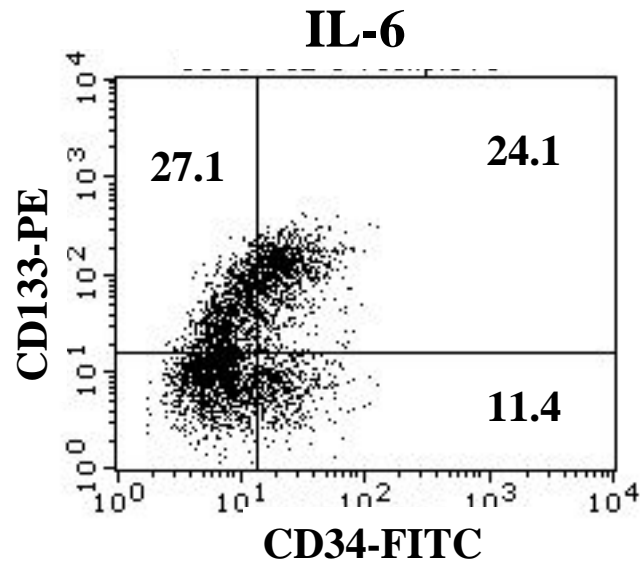**B**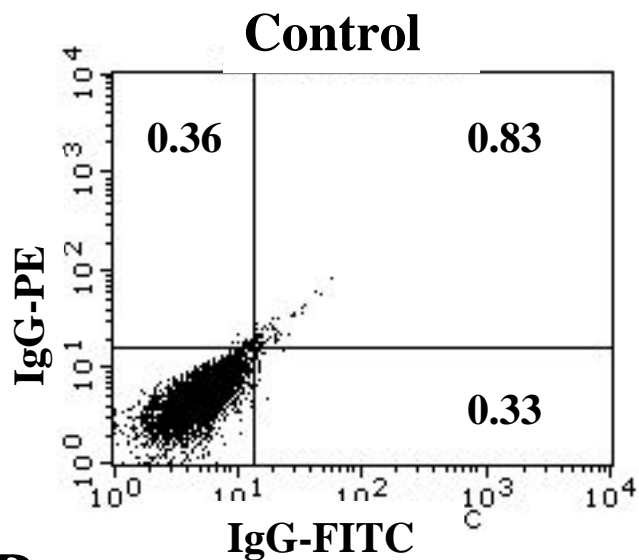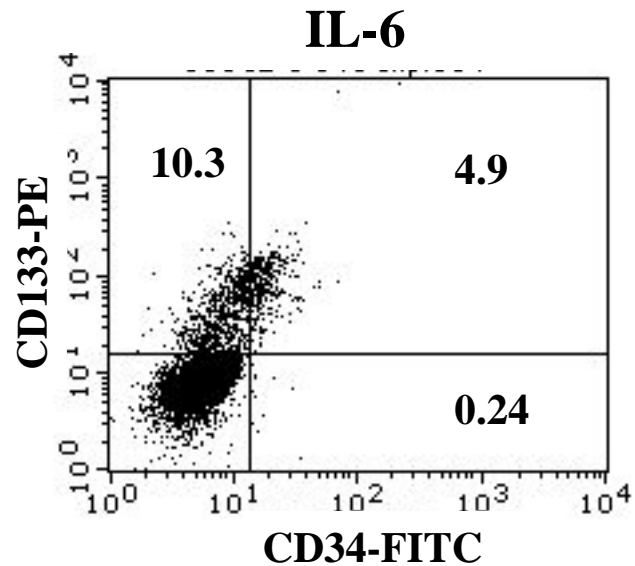

Supplement: Additional file 1 — CD133 and CD34 expression of HPC cultured in IL-6 stimulated endothelial supernatant. after one week in culture (A) CD133 and CD34 were still present, whereas a distinct subset of CD133(+) cells did not stain for CD34. Reduced CD34 was paralleled by reduced CD133 positivity in the second week (B), while still more CD133(+)CD34(-) cells than double positive cells were detected. [file 1471-2172-9-56-S1.pdf]
